# Inotodiol From *Inonotus obliquus* Chaga Mushroom Induces Atypical Maturation in Dendritic Cells

**DOI:** 10.3389/fimmu.2021.650841

**Published:** 2021-03-12

**Authors:** Perry Ayn Mayson A. Maza, Ji-Hyun Lee, Yong-Su Kim, Gyu-Min Sun, Youn-Joo Sung, Ludmila P. Ponomarenko, Valentine A. Stonik, Minsook Ryu, Jong-Young Kwak

**Affiliations:** ^1^ Department of Pharmacology, School of Medicine, Ajou University, Suwon, South Korea; ^2^ Department of Biomedical Sciences, The Graduate School, Ajou University, Suwon, South Korea; ^3^ G.B. Elyakov Pacific Institute of Bioorganic Chemistry, Far Eastern Branch of the Russian Academy of Sciences, Vladivostok, Russia; ^4^ Department of Allergy, School of Medicine, Ajou University, Suwon, South Korea; ^5^ Immune Network Pioneer Research Center, Ajou University, Suwon, South Korea; ^6^ 3D Immune System Imaging Core Center, Ajou University, Suwon, South Korea

**Keywords:** inotodiol, dendritic cells, IL-2, Chaga mushroom, atypical maturation, AKT1

## Abstract

Dendritic cells (DCs) have the ability to stimulate naïve T cells that coordinate subsequent adaptive response toward an inflammatory response or tolerance depending on the DC differentiation level. Inotodiol, a lanostane triterpenoid found in *Inonotus obliquus* (wild Chaga mushroom), is a natural compound with a wide range of biological activities. In this study, we investigated whether inotodiol promotes the maturation of bone marrow-derived DCs (BMDCs) and inotodiol-treated BMDCs induce T cell activation. Inotodiol increased the expression of surface maturation markers, including MHC-I, MHC-II, CD86, and CD40, on BMDCs without affecting the production of various cytokines, including TNF-α and IL-12p40 in these cells. T cells primed with inotodiol-treated BMDCs proliferated and produced IL-2, without producing other cytokines, including IL-12p40 and IFN-γ. Injection of inotodiol into mice induced maturation of splenic DCs and IL-2 production, and the administration of inotodiol and inotodiol-treated BMDCs induced the proliferation of adoptively transferred CD8^+^ T cells *in vivo*. The phosphatidylinositol-3-kinase inhibitor wortmannin abrogated the upregulation of Akt phosphorylation and CD86 and MHC-II expression induced by inotodiol. However, inotodiol failed to induce phosphorylation of the IκB kinase and degradation of IκB-α, and increased expression of CD86 induced by inotodiol was not blocked by an IκB kinase inhibitor. These results suggest that inotodiol induces a characteristic type of maturation in DCs through phosphatidylinositol-3-kinase activation independent of NF-κB, and inotodiol-treated DCs enhance T cell proliferation and IL-2 secretion.

## Introduction

Dendritic cells (DCs) are professional antigen-presenting cells that link innate and adaptive immune responses. DCs are activated by microbial components, such as lipopolysaccharide (LPS) (1), and cytokines, such as tumor necrosis factor (TNF)-α (2). Immature DCs express low levels of major histocompatibility complex class-II (MHC-II) surface molecules and costimulatory molecules, and produce little or no pro-inflammatory cytokines (3, 4). Upon classical maturation, DCs exhibit reduced antigen uptake, increased expression of MHC-II and costimulatory molecules, such as CD86 and CD40, the ability to produce large amounts of cytokines, and active migration to draining lymph nodes (3, 4). Matured DCs are strong inducers of T cell responses against specific pathogens (5).

Cytokine secretion by DCs is an important signal for the induction of T cell differentiation (6). Stimulated DCs secrete cytokines, where the balance between pro-inflammatory (e.g., IL-12, IL-6, and IL-1β) and immunosuppressive (e.g., IL-10) cytokines is decided by the environment (7). Immature DCs become mature effector DCs that promote the development of Th_1_, Th_2_, or regulatory T cells (8), whereas immature DCs alone and DCs without costimulatory signal and cytokine production can induce T cell anergy (9). DCs in a semi-mature state lack certain phenotypic markers or produce lower amounts of pro-inflammatory cytokines, which can lead to a tolerogenic outcome after interaction with responding T cells (10). Thus, cytokine production of activated DCs is important in generating effector responses in T cells.

*Inonotus obliquus* (*I. obliquus*), known as Chaga mushroom, is a rich source of natural compounds with a wide range of biologically active components (11–13). Based on chemical analysis of *I. obliquus*, polysaccharides, triterpenes, and polyphenols are responsible for most of its biological effects (14). One of these components is inotodiol, a lanostane-type triterpenoid that has various biological activities, including antitumor (15, 16), antiviral (17), and anti-inflammatory activities (18). Recently, inotodiol was shown to ameliorate allergy symptoms through acting on masts cells, but not on CD4^+^ T cells in a mouse model of food allergy (19). However, to our knowledge, no data are available on the immunomodulatory capacity of inotodiol or on DC modulation by its potential immunomodulatory capabilities.

In this study, we found that treatment of DCs with inotodiol increased the expression of surface markers on DCs, without affecting pro-inflammatory cytokine secretion, indicating that inotodiol-stimulated matured DCs represent a phenotype distinct from classical maturation induced by LPS.

## Materials and Methods

### Culture Medium and Reagents

DCs were cultured in RPMI-1640 medium (Sigma-Aldrich, St. Louis, MO, USA) supplemented with 10 mM sodium pyruvate, 10 μg/mL penicillin and streptomycin, HEPES buffer, 2 g/L of NaHCO_3_, 100 µM β-mercaptoethanol, and 10% fetal bovine serum (FBS) (DC culture medium, hereafter). DC culture medium was used in all *in vitro* experiments, unless otherwise stated. Recombinant mouse IL-4 and recombinant mouse granulocyte/macrophage colony-stimulation factors (GM-CSF) were purchased from JW Creagene (Seoul, South Korea). Monoclonal antibodies against MHC-I, MHC-II, CD80, CD86, and CD40 were obtained from eBioscience (San Diego, CA, USA). LPS from *Escherichia coli* (O111:b4), lanosterol, SB203580, PD98059, and LiCl were purchased from Sigma-Aldrich. Wortmannin, SB415286, SB216763, and BMS345541 were purchased from R&D Systems (Minneapolis, MN, USA).

### Purification of Inotodiol From Chaga Extract

**Figure 1:**
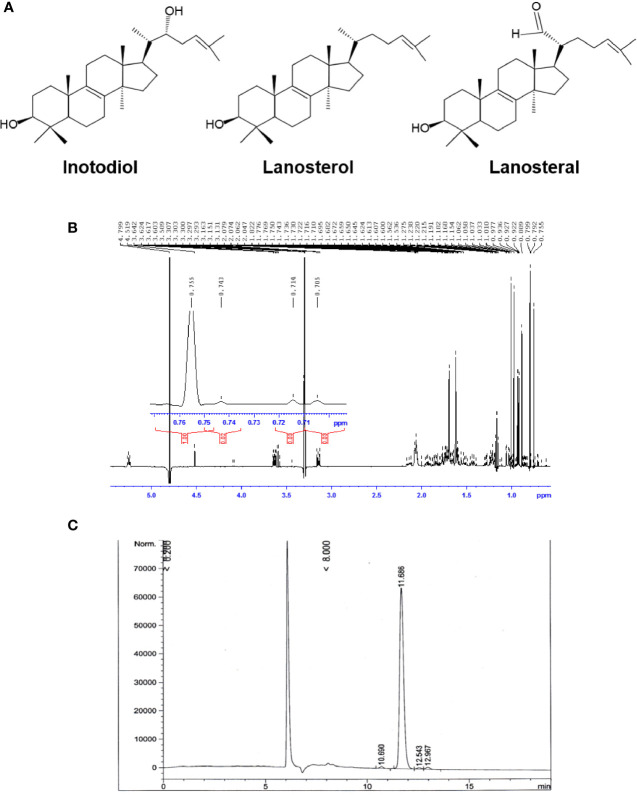
Purified inotodiol from *I obliquus*. **(A)** Chemical structures of lanosterol, inotodiol, and lanosteral. **(B)** ^1^H NMR spectrum for the identification of the chemical structure of inotodiol sample. **(C)** HPLC analysis of the purity of inotodiol.

Air-dried sclerotia of Chaga mushroom (220 g) were ground into small pieces and extracted with ethanol at room temperature for 20 h. The ethanolic extract was concentrated under reduced pressure to give 2.8 g brown amorphous solid. This material was subjected to column chromatography on silica gel (50-160 mm) (Sorbpolymer, Russia) using a hexane/ethyl acetate (5:2) system as the mobile phase. The obtained concentrate of inotodiol (1.08 g) was purified by high-performance liquid chromatography (HPLC) (Agilent 1100 chromatograph equipped with a differential refractometer) on an Ultrasphere-Si^™^ column (10 × 200 mm, 5 µm) with a hexane/ethyl acetate (5:2) system as the mobile phase (flow rate, 2 mL/min) to give pure inotodiol (320 mg) and 3β-hydroxylanosta-8,24-dien-21-al (35.3 mg) (named lanosteral in this study) (**Figure 1A**). Inotodiol and lanosteral were identified by ^1^H (**Figure 1B**) and ^13^C nuclear magnetic resonance (NMR), as reported (20, 21).

### Assessment of Inotodiol Purity

Inotodiol purity was analyzed using an Agilent 1100 chromatograph equipped with a differential refractometer on reversed phase column Supelco Ascentis RP-Amide (10 × 250 mm, 5 µm) with methanol as the mobile phase (flow rate, 2 mL/min). Inotodiol was dissolved in dimethylsulfoxide (DMSO) and stock solutions were diluted with DC culture medium.

### Preparation of Bone Marrow-Derived Dendritic Cells

C57BL/6 and BALB/c mice were maintained in accordance with institutional animal care guidelines of the Institutional Animal Care and Use Committee of Ajou University (2015-0028). Bone marrow (BM) specimens were obtained from male C57BL/6 mice. Mouse BMDCs were obtained from mouse BM precursors as described previously (22, 23). Femurs and tibiae of C57BL/6 mice were removed and the BM cavities were flushed out using 10 mL of EDTA:balanced salt solution (BSS) buffer (EDTA : BSS) with 2% FBS. After centrifugation at 1,200 rpm for 5 min, the cells were incubated with 1 mL of red cell lysis buffer (Sigma-Aldrich) under constant mixing on ice for 2 min. The cells were washed with the EDTA: BSS buffer and then re-suspended in complete DC culture medium and filtered through a 70-µM cell strainer (Falcon Corning, Arizona, USA). The cells were differentiated in complete DC culture medium supplemented with 20 ng/mL of IL-4 and GM-CSF in a 6-well culture plate with a seeding density of 3 × 10^6^ cells/mL. On the third day of incubation, the same concentration of IL-4 and GM-CSF was added and half of the DC culture medium was refreshed. Immature BMDCs were harvested on day 7 of incubation. By immunomagnetic selection of CD11c^+^ cells, BMDCs were purified to >90% using CD11c Microbeads Ultrapure (Miltenyi Biotec, Bergisch-Gladbach, Germany). Cells were identified as CD11c^+^ immature DCs based on low expression of surface MHC-II and CD80, CD86, and CD40 costimulatory molecules.

### Purification of Peritoneal Macrophages and Culture of Raw264.7 Cells

C57BL/6 mice were intraperitoneally injected with 3% thioglycolate in distilled water for 3 days. Intraperitoneal cells were isolated by lavage with phosphate-buffered saline (PBS), and macrophages were purified with anti-F4/80 antibody-bound microbeads (Miltenyi Biotec). Murine macrophage Raw264.7 cells were obtained from the Korean Cell Bank (Seoul, Korea) and were grown in RPMI-1640 medium (GIBCO/Invitrogen, Grand Island, NY, USA) containing 10% FBS supplemented with penicillin/streptomycin.

### Stimulation of Bone Marrow-Derived Dendritic Cells With Inotodiol

To investigate the effect of inotodiol, BMDCs were cultured in DC culture medium in a 24-well plate (2 × 10^5^ cells/mL) stimulated with 0.01% DMSO (vehicle control), 25 µM inotodiol, and 1 µg/mL LPS for 24 h. At the end-point of the culture, cell surface molecules were determined by flow cytometry. Cell-free supernatants were collected and stored at -80°C for the measurement of secreted cytokine level. All subsequent experiments were performed after stimulation of BMDCs with the indicated concentrations, unless otherwise stated.

### Flow Cytometry

Cells were washed with EDTA : BSS buffer, pretreated with 2% mouse serum to block nonspecific binding of antibodies, and labeled with fluorescence-conjugated antibodies against MHC-I, MHC-II, CD80, CD86, and CD40 on ice for 30 min, followed by washing with the same buffer. Dead cells were excluded using the viability dye 7-aminoactinomycin D (7-AAD) (eBioscience), and 7-AAD-negative population was then analyzed using a MACSQuant VYB flow cytometer (Miltenyi Biotec) at the 3D immune system imaging core facility of Ajou University. Flow cytometry data were analyzed using the software package FlowJo v10 (Tree Star, Ashland, OR, USA). Expression levels of MHC and costimulatory molecules were expressed as a percentage or the mean fluorescence intensity (MFI) of positively stained cells among CD11c^+^ cells.

### Cytokine Measurements

After stimulation of CD11c^+^ BMDCs (2 × 10^5^) with inotodiol (25 µM) and LPS (1 µg/mL) for 24 h, TNF-α, IL-12p40, IL-6, IL-1β, IL-2, IL-12p70, IL-4, IL-5, and interferon (IFN)-γ cytokines from the cell-free supernatant were analyzed using standard enzyme-linked immunosorbent assay (ELISA) kits (R&D Systems) with standard cytokine preparations as an internal control. Plates were read at a wavelength of 450 nm using a microplate automatic reader (BioTek, Vermont, USA). The Proteome Profiler Mouse Cytokine Array Kit (R&D Systems) was also used, per the user manual.

### MLR Assay

CD4^+^ and CD8^+^ T cells were enriched by immunomagnetic negative selection from spleens of Balb/c mice using beads labeled with CD11b, CD11c, CD19, CD45R, CD49b, CD105, MHC-II, Ter-119, and TCR/γδ (Miltenyi Biotec) and MACS separation columns (Miltenyi Biotec). Isolated CD4^+^ and CD8^+^ T cells were labeled with 10 µM carboxyfluorescein diacetate succinimidyl ester (CFSE) (Thermo Fisher Scientific, Waltham, MA, USA). BMDCs (5 × 10^3^) were treated with LPS and inotodiol in a 96-well plate (Costar, Cambridge, MA, USA) for 24 h, after which CFSE-labeled T cells (1 × 10^5^) were added at a DC/T cell ratio of 1:20 and the cells were cultured at 37^°^C in the presence of 5% CO_2_ for 4 days. Cell-free supernatants were obtained and examined for cytokine secretion. T cell proliferation was measured based on the percentage of CFSE dilution.

### *In Vitro* CD8+ T Cell Proliferation Assay

BMDCs (2 × 10^5^) were stimulated or not with inotodiol and LPS in a 96-well plate for 24 h. The BMDCs were then pulsed with 10 µM ovalbumin (OVA)_257-264_ peptide SIINFEKL (InvivoGen, San Diego, California, USA) for 1 h. Naïve CD8^+^ T cells from spleens of OT-I transgenic mice were isolated using a CD8 T cell isolation kit (Miltenyi Biotec) and were labeled with 10 µM CFSE for 10 min. After purification and labeling, the CD8^+^ T cells (1 × 10^5^) were incubated with stimulated and pulsed DCs (5 × 10^3^) at a DC/T cell ratio of 1:20 for 4 days. Then, proliferation was analyzed for CFSE dilution in proliferating T cells.

### Analysis of CD86 Expression in CD11c+ Splenic Dendritic Cells and Cytokine Production in the Blood of Inotodiol-Injected Mice

DMSO (0.01%) (control), Inotodiol (6.5 mg/kg), and LPS (250 μg/kg) were injected into the tail vein of 6-8 weeks old C57/BL6 mice. Spleens were collected and digested with DNase (20 μg/mL) and collagenase (1 mg/mL) 24 h after injection. Total cell suspensions (2 × 10^6^) of the spleen were isolated and stained with anti-CD11c, anti-CD86, anti-MHC-II, and anti-MHC-I antibodies, and the percentages of CD86^+^, MHC-II^+^, and MHC-I^+^ cells among the CD11c^+^ population were quantified using flow cytometry. For analysis of intracellular IL-2 expression, the cell surfaces of isolated splenocytes were stained with anti-CD4 (eBioscience) and anti-CD8 antibodies (eBioscience) conjugated with APC-fluorophore, and the cells were fixed using 1% paraformaldehyde. Then, cells were permeabilized using 0.1% Triton X-100 in EDTA : BSS buffer containing 2% FBS and stained with anti-IL-2 antibodies conjugated with FITC (Invitrogen). Intracellular IL-2 expression was measured in CD4^+^ and CD8^+^ cells using flow cytometry. Blood was collected into tubes containing protease inhibitors (Cell Signaling Technology, Danvers, MA, USA) 24 h after intravenous injection of inotodiol and LPS into mice, and the concentrations of IL-2 and TNF-α in the serum were quantified using ELISA kits (R&D Systems).

### *In Vivo* CD8+ T Cell Proliferation Assay

To detect antigen-specific T cell proliferation *in vivo*, CD8^+^ T cells (1 × 10^7^) from OT-I transgenic mice were labeled with 10 µM cell tracker red dye (CellTracker™ Cell Proliferation Kit, Thermo Fisher Scientific) for 20 min at 37°C and 10 µM CFSE for 30 min at 37°C, as previously described (24, 25). Cell tracker red dye was used before transfer of donor T cells to track the administered T cells in recipient mice, because it has been reported that the cell tracker dye crosses the plasma membrane of cells, where the stable and well-retained fluorescent label offers a consistent fluorescent signal (24). Fluorescence-labeled OT-I CD8^+^ T cells (5 × 10^6^ per recipient) were injected into the tail veins of C57BL/6 recipient mice (*n* = 3). *In vivo* stimulation of administered OT-I T cells was performed 16 h after cell transfer by intravenous injection of 20 µg OVA protein (Sigma Aldrich) with DMSO (0.01%), Inotodiol (6.5 mg/kg), and LPS (250 µg/kg) as a positive control in C57BL/6 recipient mice. In parallel, we also tested the *in vivo* effects of inotodiol-treated BMDCs on T cell proliferation. BMDCs (2 × 10^5^) were stimulated with inotodiol or LPS for 24 h, and the stimulated cells were pulsed with 10 µM OVA_257-264_ peptide for 1 h at 37°C. OVA-pulsed cells were then injected into the foot pad 16 h after fluorescence-labeled OT-I CD8^+^ T cells (2 × 10^6)^ were injected into recipient mice (*n* = 3). After four days, the spleen of recipient mice was recovered and the extent of proliferating T cells was measured as the percentage of CFSE-diluted cells among the cell tracker red dye-positive cells. In parallel with the T cell proliferation assay, blood was collected by cardiac puncture to quantify cytokines in the serum.

### Quantitation of Dextran Uptake

CD11c^+^ BMDCs were stimulated or not with inotodiol (25 µM) and LPS (1 µg/mL) for 24 h and then incubated with 1 mg/mL FITC-dextran (40 kDa) (Sigma-Aldrich) at 4°C (for internal control) or 37°C for 30 and 60 min. The cells were washed with EDTA : BSS buffer and labeled on ice with APC-conjugated anti-CD11c antibody. Dextran uptake was calculated as the difference in MFI between cell samples.

### Western Blotting

Levels of phosphorylated kinases, including phospho-extracellular signal-regulated kinase (ERK)-1/2 (p-ERK-1/2), phospho-p38 mitogen-activated protein kinase (MAPK) (p-p38 MAPK), phospho-Akt (p-Akt), phospho-glycogen synthase kinase (GSK)-3β (p-GSK-3β), and phospho-IκB kinase (IKK) (p-IKK), were evaluated by western blotting. When specific inhibitors of signaling pathways were used, the drugs were added 10 min before stimulation with inotodiol (25 µM) and LPS (1 µg/mL). Stimulated BMDCs were harvested and suspended in cell lysis buffer (20 mM Tris-HCl, 150 mM NaCl, 1 mM Na2EDTA, 1 mM EGTA, 1% Triton, 2.5 mM sodium pyrophosphate, and 1 mM β-glycerophosphate) containing phosphatase and proteases inhibitors (Cell Signaling Technology). Cell lysates were incubated on ice for 20 min and then centrifuged at 13,000 rpm for 10 min to obtain supernatants. Protein concentrations were measured using the Bradford assay (Bio Rad, Hercules, CA, USA). Protein concentrations were normalized and equal amounts of samples were mixed with 5× sodium dodecyl sulfate-polyacrylamide gel electrophoresis (SDS-PAGE) loading buffer (250 mM Tris-HCl, 0.25% bromophenol blue, 50% glycerol, 10% SDS, and 0.5 M dithiothreitol), heated to 95°C for 10 min, and stored at -20°C until use. Proteins (10 µg) were separated by 12% (v/v) SDS-PAGE and then transferred to nitrocellulose membranes using a wet transfer Mini-Trans Blot system (Bio Rad). The membranes were washed with 0.05% Tween-20 in PBS and subsequently incubated with blocking solution (5% bovine serum albumin and 0.05% Tween-20 in PBS) at room temperature for 1 h. Primary antibodies against p-ERK-1/2_Thr202/Tyr204_ (1:7,000), ERK-1/2 (1:4,000), p-p38 MAPK_Thr180/Tyr182_ (1:2,000), p-38 MAPK (1:4,000), p-Akt_Ser473_ (1:1,000), Akt (1:4,000), p-GSK-3β_Ser9_ (1:2,000), GSK-3β (1:4,000), p-IKK-α/β_Ser176/180_ (1:4,000), IκB-α (1:5,000), and β-actin (1:10,000) were obtained from Cell Signaling Technology. The membranes were incubated with these antibodies diluted in blocking solution as indicated at 4°C for 18 h, washed, and incubated with anti-rabbit horseradish peroxidase-linked secondary antibody (1:10,000) (Abcam, Cambridge, England) in blocking solution at room temperature for 1 h. Chemiluminescence was detected using Clarity™ western ECL substrate (Bio-Rad) followed by exposure to Medical X-ray film (AGFA, Mortsel, Belgium). Digitized images were analyzed using the ImageJ software (NIH, Bethesda, MD, USA).

### Statistical Analysis

All data are presented as the mean ± standard deviation (SD). The effect of inotodiol or LPS treatment in comparison with vehicle control was determined using Student’s *t*-test with unpaired two-sample equal variance with a two-tailed distribution in GraphPad Prism 4 (San Diego, California, USA).

## Results

### Purification and Analysis of Inotodiol

Inotodiol, a tetracyclic triterpenoid, was extracted from an air-dried Chaga mushroom and was subjected to silica gel column chromatography followed by HPLC using a normal phase column for purification. The chemical structure of the obtained inotodiol and its NMR spectra were consistent with those in previous reports (21, 26). The sample was analyzed by HPLC using a reverse-phase column. The HPLC chromatogram showed that the inotodiol obtained had 97% purity, with a retention time of 11.686 min (**Figure 1C**).

### Inotodiol Increases the Expression of MHC and Costimulatory Molecules in Bone Marrow-Derived Dendritic Cells

**Figure 2:**
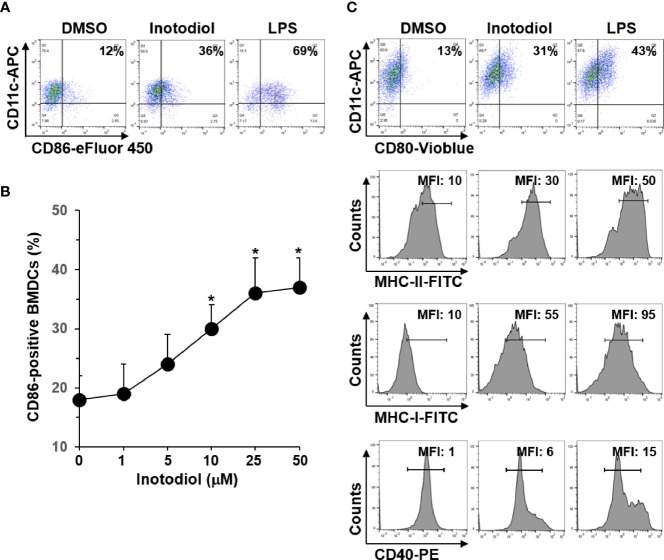
Expression levels of MHC and costimulatory molecules in inotodiol-treated BMDCs. **(A)** BMDCs (2 × 10^5^) were stimulated with DMSO (0.01%) (None), inotodiol (25 μM), or LPS (1 µg/ml) for 24 h (*n* = 3). **(B)** BMDCs were treated with the indicated concentrations of inotodiol for 24 h (*n* = 3). **(C)** BMDCs were treated as in panel A (*n* = 3). The expression levels of CD80, MHC-II, MHC-I, and CD40 in the cells were quantified by flow cytometry. Numbers represent the percentages of cells within the indicated gates among CD11c^+^ cells or MFI values. Data are presented as means ± SDs (*n* = 3). *P <0.01 versus None.

In steady state, DCs are immature and are characterized by low surface expression of MHC-I, MHC-II, and costimulatory molecules, such as CD80, CD86, and CD40 (8). To determine whether inotodiol has an effect on the maturation of DCs, we analyzed the expression level of CD86 on BMDCs using flow cytometry. As shown in **Figure 2A**, inotodiol increased CD86 expression in BMDCs to a comparable level as LPS, which is known as a DC maturation agonist. Maximum CD86 expression was obtained with 25 μM of inotodiol (**Figure 2B**). Cell viability measurements showed that inotodiol was not toxic at 50 µM (data not shown). Thus, we used the 25 µM concentration in all subsequent experiments. Inotodiol at 25 µM increased the expression of MHC-I, MHC-II, CD80, and CD40 (**Figure 2C**). As CD86 expression was increased in inotodiol-treated BMDCs (Inotodiol-BMDCs, hereafter), peritoneal macrophages also significantly increased CD86 expression on their cell surface in the presence of inotodiol (**Supplementary Figure S1A**). In addition, CD86 expression was upregulated in inotodiol-treated Raw264.7 macrophage cells (**Supplementary Figure S1B**). Taken together, these findings indicated that inotodiol upregulates the expression of MHC-II and costimulating molecules in DCs and macrophages. In contrast, lanosterol failed to upregulate the expression of CD86, MHC-II, and CD40 in BMDCs (**Supplementary Figure S2**). The related lanosterol derivative and inotodiol congener, lanosteral, also upregulated the expression of MHC-II and costimulatory molecules, although its potency was lower than that of inotodiol. Therefore, we used inotodiol rather than lanosteral as a DC-stimulating compound in this study.

### Inotodiol Induces a Mature Phenotype in Dendritic Cells

**Figure 3:**
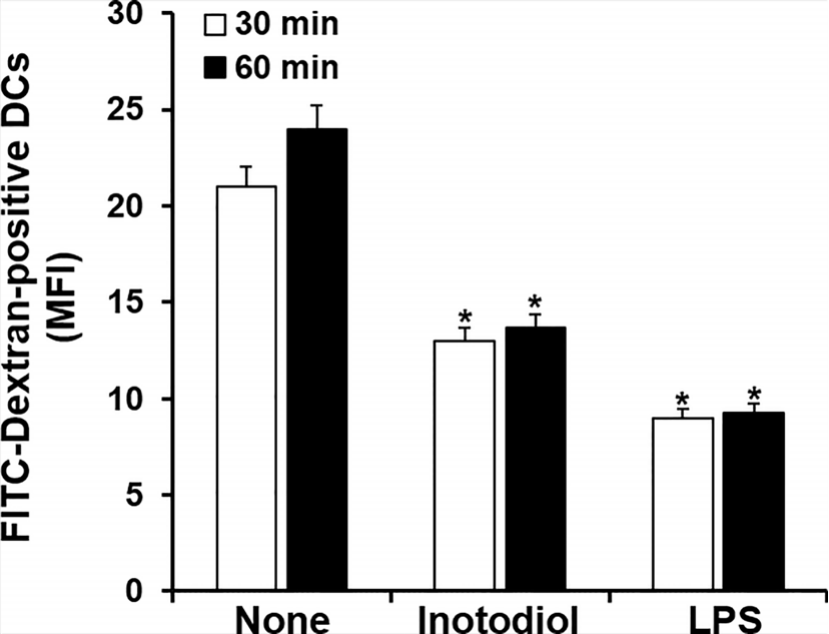
Uptake of FITC-dextran by inotodiol-treated BMDCs. BMDCs (2 × 10^5^) were stimulated with DMSO (0.01%) (None), inotodiol (25 μM), or LPS (1 µg/ml) for 24 h and were then incubated with FITC-dextran for the indicated periods at 37°C. MFI values were measured by flow cytometry. Data are presented as means ± SDs (*n* = 3). *P <0.001 versus None.

Immature DCs are characterized by a high endocytic rate (1). We next investigated changes in endocytic activity in Inotodiol-BMDCs based on the uptake of fluorescein-conjugated dextran. As shown in **Figure 3**, treatment with inotodiol or LPS for 24 h potently downregulated the uptake of FITC-dextran when compared with DMSO treatment in BMDCs, indicating that Inotodiol-BMDCs exhibited lower antigen uptake than untreated immature BMDCs. This result suggested that inotodiol induces a mature phenotype of DCs comparable with that of LPS-treated BMDCs (LPS-BMDCs, hereafter).

### Effects of Inotodiol on the Secretion of Cytokines and Chemokines in Bone Marrow-Derived Dendritic Cells

**Figure 4:**
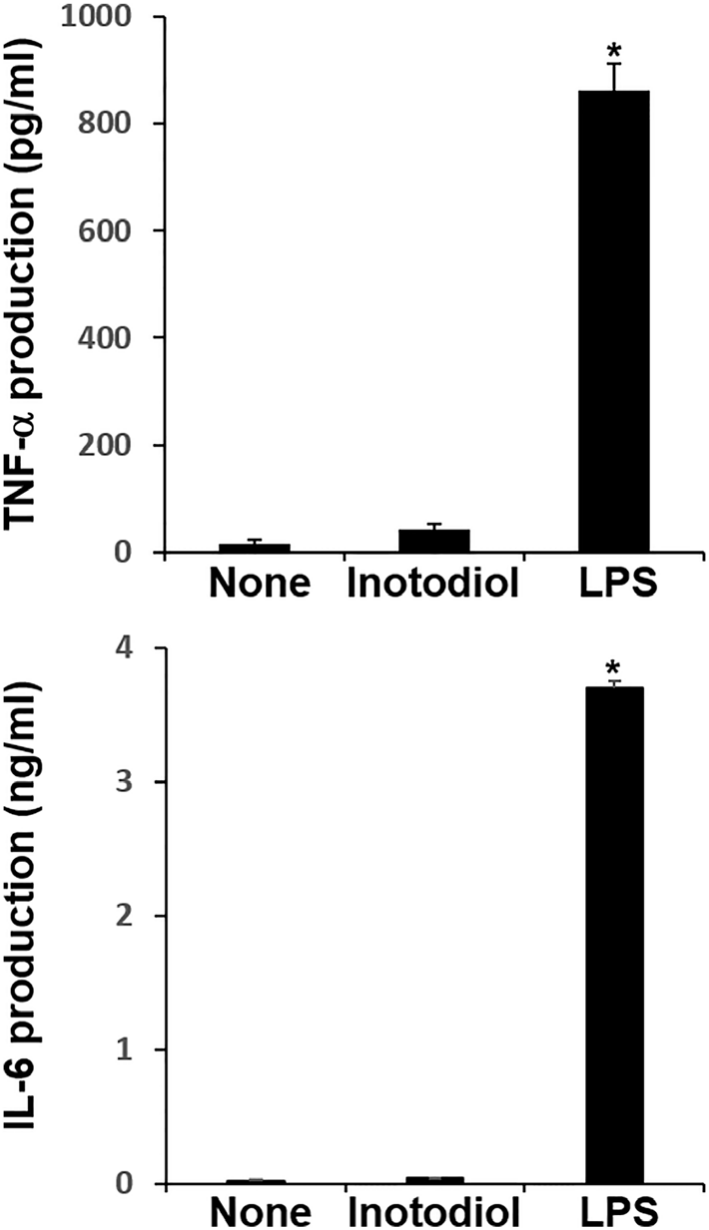
Cytokine production by inotodiol-treated BMDCs. BMDCs (2 × 10^5^) were treated with DMSO (0.01%) (None), inotodiol (25 μM), or LPS (1 µg/ml) for 24 h (*n* = 3). Cytokines secreted in culture supernatants were quantified by ELISA. Data are presented as means ± SDs (*n* = 3). *P <0.001 versus None.

We measured the levels of cytokines in supernatants collected from BMDCs cultured with inotodiol and LPS. LPS-BMDCs secreted high levels of TNF-α and IL-6, whereas Inotodiol-BMDCs produced very low amounts of these cytokines (**Figure 4**). Other pro-inflammatory and anti-inflammatory cytokines, including IL-1β, IL-10, and IL-12p40, were not produced by Inotodiol-BMDCs (data not shown). Since Inotodiol-BMDCs did not produce IL-12p40, it is possible that these cells induce a Th_2_-prone immune response. However, IL-4 and IL-5 production was not increased in Inotodiol-BMDCs (data not shown). Next, conditioned media of peritoneal macrophages cultured in the presence of inotodiol (25 μM) and LPS (1 μg/mL) for 24 h were collected, and the secretion of cytokines and chemokines was determined using a Proteome Profiler Mouse Cytokine Array Kit. The chemiluminescence signal density of each spot was quantified with ImageJ, and positive control signals were used to normalize array data (**Supplementary Figure S3A**). Analysis of the relative fold changes in expression between untreated and inotodiol-treated macrophages showed that inotodiol treatment increased the secretion of IL-1ra, CXCL2, and CCL5 and decreased that of CXCL13 compared to control, whereas LPS-stimulated macrophages secreted various cytokines and chemokines. Especially, TNF-α and IL-6 secretion was increased by LPS-treated, but not inotodiol-treated macrophages when compared to untreated control cells, as observed for cytokine production by Inotodiol-BMDCs. LPS-stimulated Raw264.7 cells also produced high levels of TNF-α and IL-6, whereas inotodiol-treated Raw264.7 cells failed to produce these cytokines (**Supplementary Figure S3B**). These results suggested that inotodiol upregulates the expression of MHC-II and CD86 without affecting the secretion of inflammatory cytokines in BMDCs, and the responses of macrophages to inotodiol are similar to those of BMDCs.

### T Cell Proliferation Induced by Inotodiol-Bone Marrow-Derived Dendritic Cells

**Figure 5:**
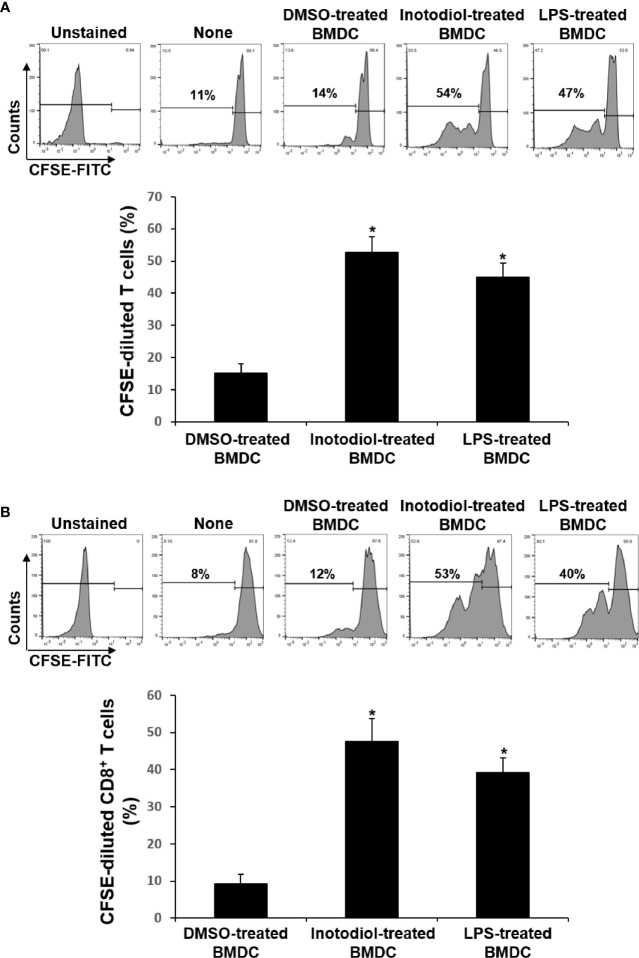
Effects of inotodiol-treated BMDCs on T cell proliferation. **(A)** BMDCs (5 × 10^3^) isolated from C57BL/6 mice were treated with DMSO (0.01%) (None), inotodiol (25 μM), or LPS (1 µg/mL) for 24 h and then cultured with CFSE-labeled T cells (1 × 10^5^) isolated from Balb/c mice for 4 days. **(B)** BMDCs (2 × 10^5^) were treated with DMSO, inotodiol, or LPS for 24 h and incubated with OVA peptide for 1 h. Stimulated BMDCs (5 × 10^3^) were cultured with CD8^+^ T cells (1 × 10^5^) from OT-I mice for 4 days. The proliferation of T cells was evaluated by measuring CFSE dilution by flow cytometry. Data are presented as means ± SDs (*n* = 3). *P <0.05 versus DMSO-treated BMDCs.

**Figure 6:**
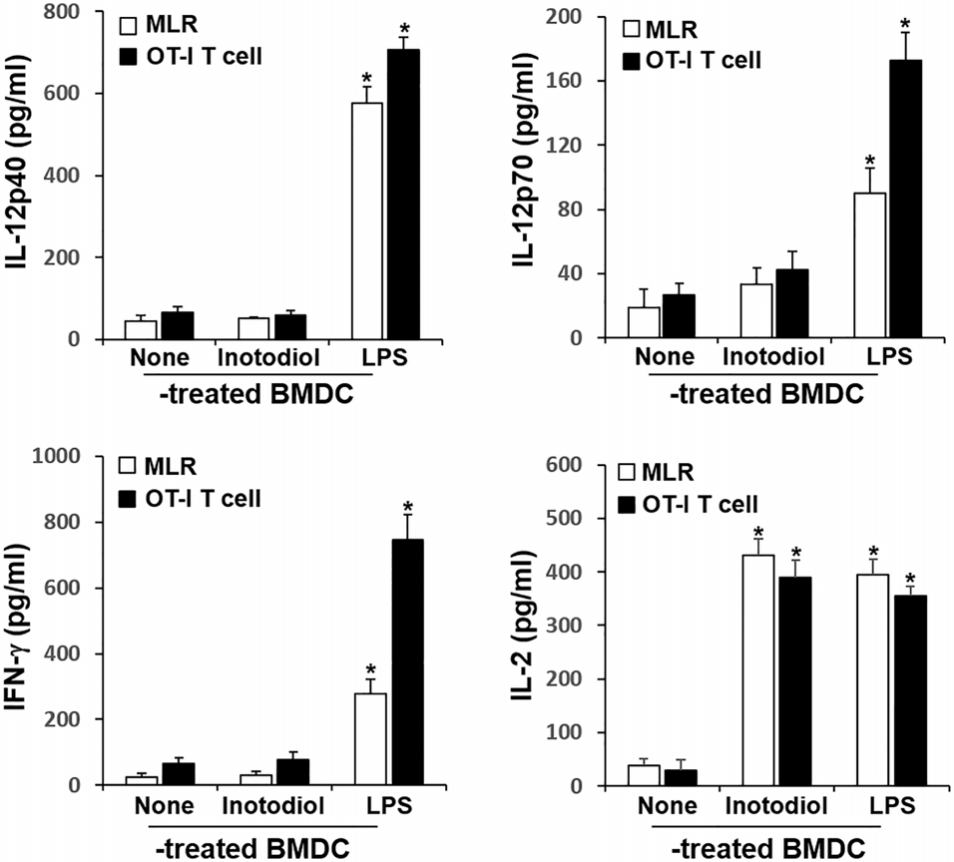
Cytokine production in cocultured inotodiol-treated BMDCs and T cells. Inotodiol- and LPS-treated BMDCs were cocultured with splenic T cells isolated from Balb/c mice (MLR) and OT-I CD8^+^ T cells (OT-I T cell) for 4 days, as in **Figure 6**. The secretion of cytokines was measured by ELISA. Data are presented as means ± SDs (*n* = 3). *P <0.001 versus DMSO-treated BMDCs.

The major role of mature DCs is to induce antigen-specific T cell activation and proliferation, depending on the nature of the DC stimuli (6). Inotodiol promotes atypical maturation of DCs, with upregulation of MHC-I, MHC-II, and costimulatory molecules, but without pro-inflammatory cytokine production. Thus, we next determined the capacity of BMDCs to stimulate T cells. As shown in **Figure 5A**, Inotodiol-BMDCs that had been stimulated with inotodiol for 1 day and cocultured with splenic T cells for 4 days increased the proliferation of splenic T cells in MLR when compared to untreated control BMDCs. The degree of T cell proliferation induced by Inotodiol-BMDCs was similar to that induced by LPS-BMDCs in the MLR assay. Next, BMDCs were stimulated by adding inotodiol or LPS for 24 h, washed, and pulsed with soluble OVA peptide SIINFEKL. The stimulated BMDCs were cultured with OT-I CD8^+^ T cells (DC:T cell ratio = 1:20) for 4 days. As shown in **Figure 5B**, OVA peptide-pulsed Inotodiol-BMDCs more strongly induced OT-I T cell proliferation than unstimulated BMDCs. Likewise, CD8^+^ T cells from OT-I mice underwent antigen-specific proliferation when cocultured with OVA peptide-pulsed LPS-BMDCs. Next, we examined T cell cytokine profile after 4 days of cultivation with Inotodiol-BMDCs. CD8^+^ T cells activated by Inotodiol-BMDCs did not secrete IL‐12p40, IL-12p70, and IFN-γ, whereas CD8^+^ T cells activated by LPS-BMDCs secreted high levels of these cytokines as compared to untreated cells (**Figure 6**). In addition, CD8^+^ T cells activated by Inotodiol-BMDCs secreted very low amounts of IL‐4, IL-5, and IL-10 when compared to those activated by LPS-BMDCs (data not shown). However, OT-I T cell stimulated with Inotodiol-BMDCs secreted a significant amount of IL-2 in an OVA-specific reaction. Similar to OVA-specific presentation, CD4^+^ and CD8^+^ T cells in MLR showed significantly induced IL-2 secretion. T cells incubated with inotodiol alone did not proliferate or secrete IL-2, excluding a mitogenic effect of inotodiol on T cells and Inotodiol-BMDCs alone did not secrete IL-2 (data not shown). Taken together, these results suggested that inotodiol induces DC activation without secretion of inflammatory cytokines, and Inotodiol-BMDCs promote T cell proliferation through the production of IL-2 alone.

### Effects of Inotodiol on Splenic Dendritic Cell Maturation and IL-2 Production *In Vivo*

**Figure 7:**
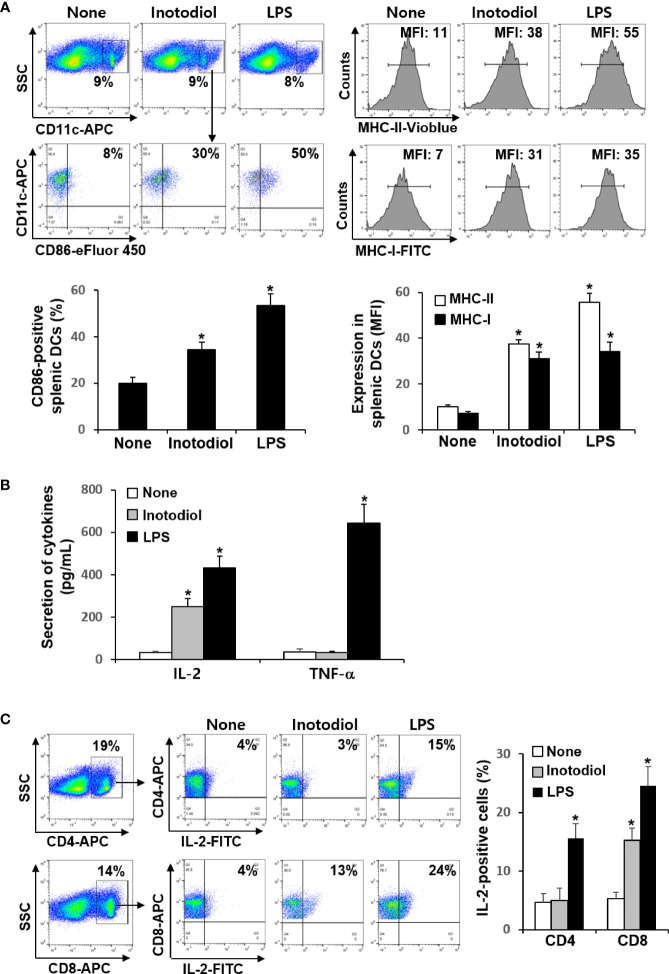
CD86 expression and cytokine production in inotodiol-injected mice. C57BL/6 mice were injected intravenously with DMSO (0.01%) (None), inotodiol (6.5 mg/kg), or LPS (250 µg/kg) (*n* = 3). Total splenocytes and blood were isolated 24 h after injection. **(A)** CD11c^+^ DCs were gated from splenocytes, and the expression levels of CD86, MHC-II, and MHC-I in the CD11c^+^ cells were quantified using flow cytometry. Numbers represent the percentages of cells within the indicated gates of cells or MFI values in CD11c^+^ cells. **(B)** The concentrations of IL-2 and TNF-α in the serum were measured by ELISA. **(C)** CD4^+^ and CD8^+^ cells were gated from total splenocytes, and intracellular expression of IL-2 in the gated cells was quantified by flow cytometry. Data are presented as means ± SDs (*n* = 3). *P <0.01 versus None.

We investigated whether inotodiol increases CD86 and MHC expression in splenic DCs *in vivo*. Total cell suspensions of spleens collected 24 h after injection of inotodiol (6.5 mg/kg) and LPS (250 μg/kg) into the tail vein were stained with anti-CD11c and anti-CD86 antibodies, and the percentages of CD86^+^ cells among CD11c^+^ cells were quantified using flow cytometry (**Figure 7A**). Intravenous injection of inotodiol resulted in upregulated CD86 expression on splenic CD11c*^+^* DCs, although CD86 expression was lower in inotodiol-treated mice than in LPS-treated mice. We also observed increased expression of MHC-I and MHC-II in splenic CD11c^+^ DCs of inotodiol-injected mice and LPS-injected mice. *In vitro* coculture of DCs and T cells in the presence of inotodiol resulted in the production of IL-2 alone without the production of proinflammatory cytokines, including TNF-α. Thus, we measured the concentrations of IL-2 and TNF-α in the serum 24 h after injection of inotodiol and LPS into the tail vein. As shown in **Figure 7B**, IL-2 production was significantly increased upon injection of inotodiol (248 ± 37 pg/mL) or LPS (431 ± 55 pg/mL), compared to the control (*P <*0.01). However, an increase in TNF-α production was observed after the injection of LPS, but not of inotodiol. Next, splenic cells of inotodiol-injected mice were gated into CD4^+^ and CD8^+^ cells, and the intracellular expression levels of IL-2 were measured by flow cytometry. IL-2^+^ cells were significantly increased in CD8^+^ cells but not in CD4^+^ cells (**Figure 7C**). In comparison, LPS injection increased the number of IL-2^+^ cells in both CD4^+^ and CD8^+^ cells. These results suggest that inotodiol-induced IL-2 production *in vivo* is mediated by CD8^+^ T cells.

### Effects of Inotodiol and Inotodiol-Bone Marrow-Derived Dendritic Cells on Transferred CD8+ T Cells *In Vivo*

**Figure 8:**
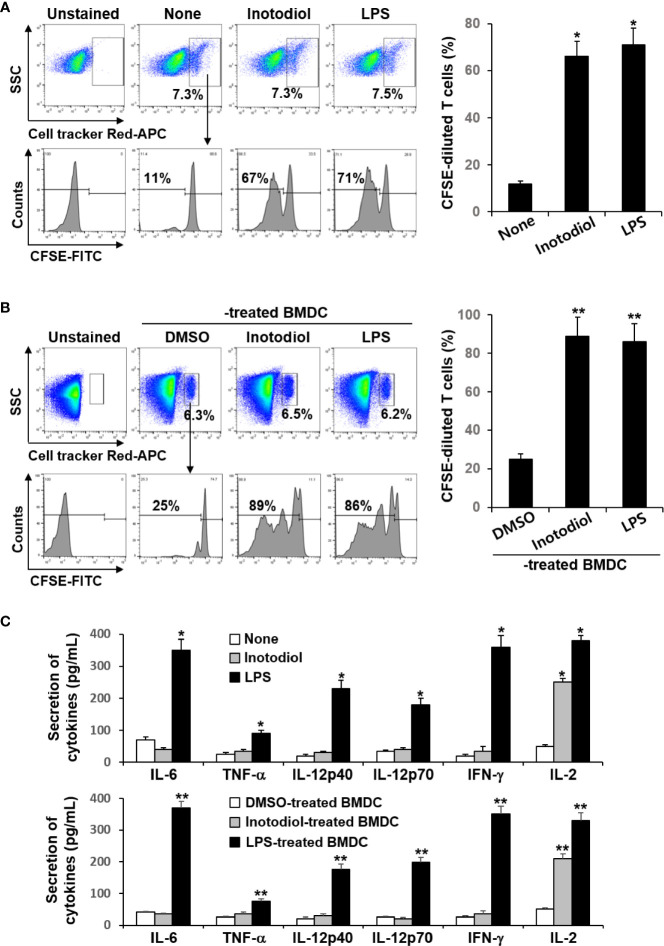
Proliferation of transferred OT-I CD8^+^ T cells and cytokine production after injection of inotodiol and Inotodiol-BMDCs *in vivo*. CD8^+^ T cells (5 × 10^6^) from OT-I mice were isolated, labeled with cell tracker red dye and CFSE, and then injected into mice as described in Materials and Methods. **(A)** OVA protein with DMSO (0.01%) (None), inotodiol (6.5 mg/kg), or LPS (250 µg/kg) were injected intravenously into recipients with transferred OT-I CD8^+^ T cells (*n* = 3). **(B)** BMDCs (2 × 10^5^) were treated with DMSO (0.01%) (DMSO-treated BMDC), inotodiol (25 μM) (Inotodiol-treated BMDC), or LPS (1 µg/ml) (LPS-treated BMDC) for 24 h and incubated with OVA peptide for 1 h, as shown in **Figure 6**, and injected into recipient mice with transferred OT-I CD8^+^ T cells (2 × 10^6)^ (*n* = 3). On day 4 after injection of inotodiol or Inotodiol-BMDCs, splenocytes were isolated from recipient mice and the proliferation of transferred T cells was evaluated by measuring CFSE dilution among the cell tracker-stained cells using flow cytometry. **(C)** Blood was collected from recipient mice as described in panels **(A)** (in the upper panel) and **(B)** (in the lower panel) on day 4 after injection, and the concentration of cytokines in the serum was measured by ELISA (*n* = 3). Numbers represent the percentages of cells within the indicated gates of the cells. Data are presented as means ± SDs (*n* = 3). *P <0.05 versus None. **P <0.05 versus DMSO-treated BMDC.

Donor CD8^+^ T cells from OT-I mice were labeled with fluorescent cell tracker red dye and CFSE and then injected intravenously into recipient mice, followed by injection of OVA protein and inotodiol. LPS was used as a positive control. The extent of CFSE dilution of proliferating CD8^+^ T cells among the cell tracker-stained population in isolated splenocytes from recipient mice was measured at 4 days after adoptive transfer of T cells. It has been reported that the cell tracker red dye has cell retention characteristics with bright fluorescence intensity even after 72 h of cell staining (24). Flow cytometry analysis showed that the percentage of cell tracker-positive cells in total splenocytes isolated from DMSO, inotodiol, and LPS-injected mice was not different (**Figure 8A**). Administration of inotodiol significantly increased CFSE dilution in cell tracker-stained OT-I CD8^+^ T cells when compared to control. The degree of T cell proliferation induced by inotodiol injection was not significantly different from that induced by LPS injection. We next assessed whether administration of BMDCs, which were atypically matured by inotodiol in *in vitro* cultures, induced T cell proliferation *in vivo*. Therefore, we transferred the cell tracker red dye and CFSE-labeled OT-I CD8^+^ T cells into C57BL/6 mice, followed by injection of OVA peptide-primed Inotodiol-BMDCs and LPS-BMDCs 16 h later. At day 4 after injection of Inotodiol-BMDCs, T cell proliferation was examined by analyzing CFSE dilution in cell tracker-stained cells (**Figure 8B**). The administration of Inotodiol-BMDCs and LPS-BMDCs induced the proliferation of transferred OT-I CD8^+^ T cells. Finally, we investigated whether inotodiol and Inotodiol-BMDCs induce cytokine production in the blood of mice with transferred OT-I CD8^+^ T cells. OVA protein and inotodiol-injected mice showed elevated serum levels of IL-2 compared to control mice (**Figure 8C**). In contrast, other cytokine levels, including IL-6, TNF-α, IL-12p40, IL-12p70, and IFN-γ, were not significantly increased in serum obtained from inotodiol-injected mice, whereas these cytokine levels were significantly increased in serum obtained from LPS-injected mice, compared to control mice. We also observed similar cytokine production in blood collected on day 4 after intravenous injection of Inotodiol-BMDCs and LPS-BMDCs into mice.

### Effects of Akt and IκB Kinase Inhibitors on the Expression of MHC-II and CD86 in Inotodiol-Bone Marrow-Derived Dendritic Cells

**Figure 9:**
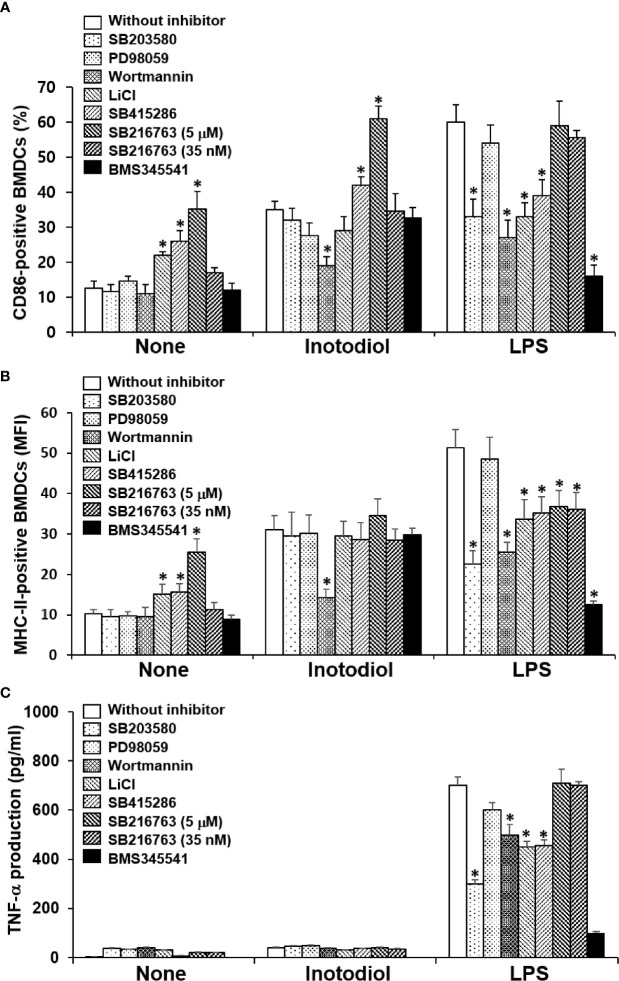
Effects of kinase inhibitors on the expression of CD86 and MHC-II and the production of TNF-α in inotodiol-treated BMDCs. BMDCs (2 × 10^5^) were pretreated or not with various kinase inhibitors, including SB203580 (5 µM), PD98059 (10 µM), Wortmannin (1 µM), LiCl (10 mM), SB415286 (10 µM), SB216763 (5 µM and 35 nM), and BMS345541 (1 µM) for 1 h and then cultured with DMSO (0.01%) (None), Inotodiol (25 µM), or LPS (1 µg/mL) for 24 h. The expression of CD86 **(A)** and MHC-II **(B)** was measured by flow cytometry. **(C)** The secretion of TNF-α was measured by ELISA. Data are presented as means ± SDs (*n* = 3). *P <0.05 versus without kinase inhibitor.

p38 MAPK, Akt, and NF-κB are known to be responsible for LPS-induced maturation (27, 28). To investigate the molecular mechanism by which inotodiol induces atypical maturation of DCs, specific kinase inhibitors, including p38 MAPK inhibitor (SB203580), phosphatidylinositol-3-kinase (PI3K) inhibitor (wortmannin), and IKK inhibitor (BMS345541) (29), were employed. BMDCs were pretreated with the kinase inhibitors for 10 min and then stimulated by inotodiol or LPS for 24 h. As shown in **Figure 9A**, SB203580 and BMS345541 abrogated the upregulation of CD86 expression in LPS-BMDCs, but not in Inotodiol-BMDCs, whereas wortmannin significantly inhibited the upregulation of CD86 expression in both Inotodiol-BMDCs and LPS-BMDCs. GSK-3 is also known to control the maturation and cytokine production of DCs (30, 31). Phosphorylation of GSK-3α at Ser_21_ and of GSK-3β at Ser_9_ by activated Akt inhibits the kinase activity of GSK-3 (32). Thus, the effects of GSK-3β inactivation on CD86 expression were evaluated by using a pharmacological inhibitor, LiCl and specific inhibitors, SB415286 and SB216763 (33). SB415286 (10 μM) and SB216763 (10 μM) alone as well as LiCl, significantly upregulated CD86 expression in unstimulated BMDCs, consistent with the previous findings (34). However, SB216763 at the IC_50_ concentration (35 nM) for GSK-3 had no effect on the expression of CD86 in both Inotodiol-BMDCs and LPS-BMDCs. SB216763 at a high concentration of 10 μM, but not at a low concentration of 35 nM exhibited the additive effect on CD86 expression in Inotodiol-BMDCs. **Figure 9B** shows that MHC-II expression in Inotodiol-BMDCs was also inhibited by wortmannin, but not by the other kinase inhibitors. However, wortmannin had no effect on the production of TNF-α in Inotodiol-BMDCs, whereas it blocked the increase in TNF-α production in LPS-BMDCs (**Figure 9C**). In addition, neither SB216763 nor LiCl affected TNF-α production in Inotodiol-BMDCs.

### Akt Phosphorylation in Inotodiol-Bone Marrow-Derived Dendritic Cells

**Figure 10:**
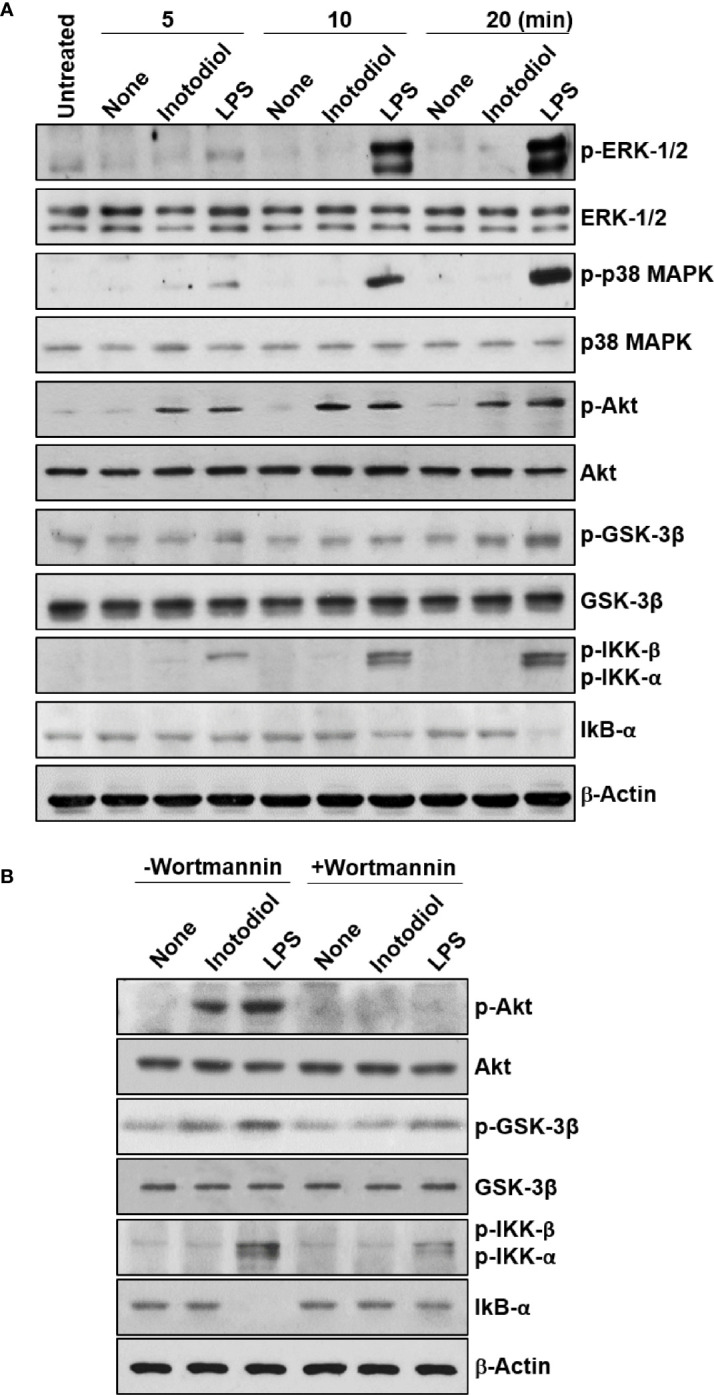
Akt and GSK-3β phosphorylation in inotodiol-treated BMDCs. **(A)** BMDCs (2 × 10^6^) were treated with DMSO (0.01%) (None), inotodiol (25 µM), or LPS (1 µg/ml) for the indicated periods. The samples were then subjected to western blot analysis using anti-phosphorylated antibodies. β-Actin was used as loading control. **(B)** BMDCs (2 × 10^6^) were pretreated with (+Wortmannin) or not (−Wortmannin) with wortmannin (100 nM) for 10 min and then stimulated with DMSO (0.01%) (None), inotodiol (25 µM), or LPS (1 µg/ml) for 20 min. Samples were subjected to Western blot analysis, as in panel **(A)**. Data represent four independent experiments.

We used western blotting to detect the phosphorylated forms of the signaling molecules, p38 MAPK, Akt, and NF-κB (28). LPS-BMDCs, but not Inotodiol-BMDCs, showed increased phosphorylation of ERK-1/2 and p38 MAPK (**Figure 10A**). The expression of p-IKK-α/β, NF-κB signaling pathway proteins, was also significantly increased in LPS-BMDCs, but not Inotodol-BMDCs. Activated IKKs can rapidly phosphorylate IκB-α, leading to its ubiquitination and proteasomal degradation (35). LPS, but not inotodiol, induced a significant decrease in IκB-α protein after 20 min of cell stimulation. In comparison, inotodiol and LPS induced substantial increases in p-Akt_Ser473_ and p-GSK-3β_Ser9_ proteins in a time-dependent manner, whereas they had no effect on total Akt and GSK-3β. Next, we tested whether a PI3K inhibitor would abrogate protein phosphorylation in Inotodiol-BMDCs and LPS-BMDCs. The increases in p-Akt and p-GSK-3β in Inotodiol-BMDCs and in p-Akt and p-IKK-α/β in LPS-BMDCs were diminished upon pretreatment of the cells with wortmannin, whereas the level of p-GSK-3β in LPS-BMDCs was not significantly affected by wortmannin (**Figure 10B**). As SB216763 at 5 μM alone increased CD86 expression in BMDCs, we tested whether wortmannin would alter CD86 expression in SB216763-treated BMDCs. As shown in **Supplementary Figure S4A**, wortmannin failed to block the increase in CD86 expression induced by SB216763 alone. In addition, the increase in GSK-3β phosphorylation in the presence of SB216763 was not affected by wortmannin (**Supplementary Figure S4B**), suggesting that activation of BMDCs by SB216763 at a high concentration is not related to the PI3K/Akt pathway. Thus, among the kinase inhibitors tested, the PI3K inhibitor caused both inhibition of Akt phosphorylation and upregulation of CD86 expression in Inotodiol-BMDCs, suggesting that inotodiol activates the PI3K/Akt pathway, but not the p38 MAPK and NF-κB pathways, to induce CD86 expression on BMDCs.

## Discussion

Inotodiol is a lanosterol hydroxylated at carbon 22 (26, 36). In this study, inotodiol, but not lanosterol significantly increased MHC-II and CD86 expression in both DCs and macrophages, although the increases in MHC-I, MHC-II, CD86, and CD40 on Inotodiol-BMDCs were lower than those on LPS-BMDCs. We used lanosteral, an inotodiol congener and a lanosterol derivative with aldehyde at carbon 21. Lanosteral also significantly increased MHC-II and CD86 expression in DCs, but its effects were not comparable to those of inotodiol. The relationship between the structure and function of lanostane triterpenes and the action mechanism of inotodiol have not been deciphered. Among three lanostane triterpenoids, inotodiol alone was reported to inhibit cancer cell proliferation (16). In comparison, inotodiol and lanosterol reportedly increased tyrosinase activities as well as melanin content in B16 melanoma cells (37). Further, it has been shown that the activity of another triterpene lanostane, ganoderic acid, on the growth and invasiveness of cancer cells is linked to the hydroxylation of its structure, as the inactive compound is not hydroxylated (38), suggesting that hydroxylation of lanosterol may be responsible for its potent immunological activities. However, the accumulation of endogenous lanosterol, the first sterol in cholesterol biosynthesis, in macrophages has been shown to exert antimicrobial and anti-inflammatory activities, suggesting that lanosterol is an endogenous mediator of innate immune responses (39). Thus, we cannot rule out the possibility that cholesterol metabolism is affected by exogenous addition of inotodiol, which has been only detected in Chaga mushroom. Alternatively, triterpenoids, including lanostane, may affect membrane fluidity (39).

LPS-stimulated mature DCs produce large amounts of pro-inflammatory and immunomodulatory cytokines (40). In this study, Inotodiol-BMDCs failed to produce IL-1β, IL-6, IL-10, IL-12p40, and TNF-α, whereas LPS-BMDCs produced substantial amounts of these cytokines. In addition, the low TNF-α production in inotodiol-stimulated BMDCs was not upregulated by GSK-3 inhibitors and other kinase inhibitors. These results suggest that inotodiol-induced maturation of BMDCs is independent of pro-inflammatory cytokines. In line with these findings, loss of E-cadherin adhesion stimulated DCs to upregulate the expression of costimulatory molecules and MHC-II, but failed to induce immunostimulatory cytokine release (34). Gotz et al. reported that *Plasmodium falciparum*-infected RBCs also induced an unusual phenotype of DCs with upregulated costimulatory molecules, but without secretion of cytokines typical of inflammatory responses, such as IL-1β, IL-6, IL-10, and TNF-α (41). In addition, the genes predominantly activated by *P. falciparum-*infected RBCs were mainly involved in lipid synthesis-related pathways, such as cholesterol biosynthesis (41). Thus, atypical maturation of DCs can be induced by cellular conditions, certain infectious agents, and natural products, including inotodiol.

The increased expression of cell surface molecules in matured DC, including MHC and costimulatory molecules, is essential for the priming of naïve T cells (42). The subsequent adaptive response toward an inflammatory response or tolerance depends on the production of cytokines. In this study, T cells primed by either Inotodiol-BMDCs in MLR or OVA-pulsed Inotodiol-BMDCs produced only IL-2, but not IL-12p40, IL-12p70 and IFN-γ. IL-2 is a key factor in driving the proliferation of activated T cells (43, 44). Thus, Inotodiol-BMDCs are implicated in inducing IL-2 secretion, leading to T cell proliferation. The production of IL‐2 is largely restricted to the activated CD4^+^ T cells (45), but activated CD8^+^ T cells and DCs also have been reported to secrete IL‐2, albeit at lower levels than activated CD4^+^ T cells (46, 47). Contact of CD4^+^ T cells with DCs activates these T cells and leads to a re-production of IL-2 (48). However, IL‐2 in concert with Th-polarizing cytokines produced by T cells and DCs can prime naïve Th cells for differentiation into different Th-cell lineages (49). This study showed IL-2 production without polarizing cytokines through coculture of Inotodiol-BMDCS and T cells, suggesting that the differentiation of the cocultured T cells into a specific subtype may be induced in a cytokine-dependent manner. The increase in IL-2 production can be explained by the finding that a significant upregulation in MHC-I, MHC-II, and costimulatory molecules on Inotodiol-BMDCs led to the mature form of DCs. T_reg_ cells also seem to be recipients of IL-2 signals when conventional T cells responding to self or other antigens produce IL-2 (50). Tolerogenic T cells are induced by semi-mature DCs, and these T cells release immunosuppressive cytokines, such as IL-10 and TGF-β (51). However, IL-10 and TGF-β production was not increased by coculture of T cells and Inotodiol-BMDCs (data not shown). Moreover, Inotodiol-BMDCs and T cells cocultured with Inotodiol-BMDCs were unable to express Foxp3 (data not shown).

Inotodiol may induce DC maturation through direct action on the cells or *via* the release of pro-inflammatory cytokines, including TNF-α, in autocrine and paracrine patterns (52). In this study, Inotodiol-BMDCs failed to release TNF-α, IL-1β, and other cytokines, suggesting that DC maturation was induced through direct action of inotodiol on the cells. DC activation can be induced *via* various signaling pathways. As observed for cytokine production, distinct signaling pathways were observed in inotodiol-treated DCs. Western blot analysis revealed that LPS stimulation induced the activation of ERK-1/2, p38 MAPK, and NF-κB, as well as Akt activation. The selective inhibitor of p38 MAPK, SB203580, inhibited CD86 expression in LPS-BMDCs, but not Inotodiol-BMDCs. Moreover, LPS, but not inotodiol phosphorylated p38 MAPK in BMDCs. These results suggest that inotodiol-induced maturation of BMDCs is independent of p38 MAPK activation. DC maturation has been reported to occur through the inhibition of GSK-3β (30). However, in this study, the GSK-3β inhibitors tested had distinct effects on inotodiol-induced upregulation of CD86 and MHC-II expression. SB415286 and SB216763 are specific, potent, and selective cell-permeable, ATP-competitive inhibitors of GSK-3 (33). In comparison, LiCl is known to inhibit GSK-3 activity by enhancing PI3K/Akt activity (53) and by replacing Mg ions at a site distinct from the ATP-binding site of GSK-3 (54). SB216763 alone enhanced CD86 expression, as reported previously (30), and augmented CD86 expression in Inotodiol-BMDCs, whereas LiCl and SB415286 had no significant effects on CD86 expression in Inotodiol-BMDCs. It has been shown that enzymatically active GSK-3 acts as a crucial positive regulator of pro-inflammatory cytokines (e.g., TNF-α, IL-1β, and IL-6) (55). However, TNF-α and IL-1β production was not affected by SB216763 at various concentrations (data not shown). Thus, it can be speculated that GSK-3 is not directly stimulated by inotodiol. Activated PI3K induces phosphorylation of Akt, which then acts upon its extensive range of targets (56). We found that Akt phosphorylation was induced after inotodiol treatment, and the effect on inotodiol-induced CD86 expression and Akt phosphorylation was strongly diminished by wortmannin, indicating that inotodiol-induced maturation may partially depend on the activation of this pathway. The PI3K pathway generally promotes unconventional DC maturation (57). PI3K knockout mice exhibited elevated Th_1_ responses, including IL-12 production, suggesting that PI3K/Akt may act by exerting an effect on counter-regulatory circuits in the production of cytokines (58).

In the cytoplasm, NF-κB exists bound to IκB. Following cellular stimulation with specific inducers, IκB is phosphorylated by the IKK complex and degraded by the proteasome, and then, NF-κB translocates to the nucleus, where it regulates the transcription of several genes (58). Analysis with IKK inhibitors suggested that robust NF-κB activation is dispensable for the canonical features of steady-state DC maturation (59). In this study, inotodiol did not cause evident changes in the NF-κB pathway, which is well known to affect conventional DC maturation in response to LPS. It has been shown that inotodiol has no effect on LPS-induced activation of NF-κB in Raw264.7 cells (60). In addition, inotodiol failed to elicit significant increases in any of the cytokines tested, whereas LPS-induced production of pro-inflammatory cytokines, including TNF-α, IL-1β, and IL-6 is related to NF-κB signaling. In contrast to its effects on CD86 and MHC-II expression, the effects of inotodiol on cytokine production could not be explained by Akt and GSK-3 phosphorylation, as inhibitors of these pathways had no effects on cytokine production by Inotodiol-BMDCs. Based on these findings, we speculate that PI3K/Akt upregulates CD86 and MHC-II expression, without affecting pro-inflammatory cytokine production, independent of NF-κB in Inotodiol-BMDCs.

In this study, we observed *in vitro* effects of inotodiol on DC maturation and T cell proliferation, and the injection of inotodiol into mice induced the maturation of splenic DCs and IL-2 secretion in the blood. In addition, the administration of Inotodiol-BMDCs induced the proliferation of adoptively transferred CD8^+^ T cells in recipient mice and IL-2 production without the production of other cytokines, including TNF-α and IL-12p40. We also observed increased expression of intracellular IL-2 in CD8^+^ T cells after injection of inotodiol *in vivo*, suggesting that this differential response in IL-2 production between CD4^+^ and CD8^+^ T cells may be caused by atypically matured DCs, although the immunological role of this type of DCs *in vivo* is not known yet. IL-2-producing CD8^+^ T cells have been suggested to have a potent expansion capacity and form long-lasting memory (61). IL-2 production by tumor-infiltrating CD8^+^ T cells is related to antitumor immunity (62), and IL-2 produced by CD8^+^ T cells can stimulate Tregs (63). However, Orozco Valencia et al. suggested that low IL-2 doses are effective in the treatment of autoimmune diseases, whereas high doses of IL-2 are effective against melanomas because high doses of IL-2 promote an imbalance in the immune system regulation by increasing the proliferation of effector T-cells (64). Thus, further studies are needed to investigate whether inotodiol and inotodiol-activated DCs may exert immunological effects in disease models, including cancer and autoimmune diseases.

In conclusion, this study demonstrated atypical maturation of DCs by inotodiol, as classical maturation of DCs typically involves the upregulation of surface markers and pro-inflammatory cytokine secretion. Atypical maturation of Inotodiol-BMDCs efficiently induced proliferation of T cells and IL-2 secretion, without production of other cytokines.

## Data Availability Statement

The original contributions presented in the study are included in the article/**Supplementary Material**. Further inquiries can be directed to the corresponding authors.

## Ethics Statement

The animal study was reviewed and approved by the Institutional Animal Care and Use committee of Ajou University (IACUC approval number 2015-0028).

## Author Contributions

PM, J-HL, and Y-SK performed the immunological experiments. G-MS and Y-JS helped with the *in vivo* experiments. LP and VS purified the inotodiol. MR and J-YK supervised the research and wrote the paper. All authors contributed to the article and approved the submitted version.

## Funding

This work was supported by a grant of the Korea Health Technology R&D Project through the Korea Health Industry Development Institute (KHIDI), funded by the Ministry of Health & Welfare, Republic of Korea (HI16C0992 and HR16C0001), and by the Basic Science Research Capacity Enhancement Project through the Korea Basic Science Institute (National Research Facilities and Equipment Center) grant funded by the Ministry of Education (2019R1A6C1010003).

## Conflict of Interest

The authors declare that the research was conducted in the absence of any commercial or financial relationships that could be construed as a potential conflict of interest.

## Supplementary Material

Supplementary Figure 1 Inotodiol increases CD86 expression in macrophages and Raw264.7 cells *in vitro*. **(A)** Peritoneal macrophages (2 × 10^5^/mL) isolated from C57BL/6 mice were purified with anti-F4/80 antibody-bound microbeads and treated with DMSO (0.01%) (None), inotodiol (25 μM), or LPS (1 µg/mL) for 24 h. CD86 expression was measured by flow cytometry using an anti-CD86 antibody conjugated with eFluor 450. Representative histograms show CD86 expression in each group (*n* = 3). **(B)** Raw 264.7 cells (2 × 10^5^/mL) were treated with DMSO (0.01%) (None), inotodiol (25 μM), or LPS (1 µg/mL) for 24 h. CD86 expression was determined by flow cytometry. Data are presented as means ± SDs (*n* = 3). **p <*0.05, versus None.

Supplementary Figure 2 Effects of lanosterol, inotodiol, and lanosteral on MHC-II, CD86, and CD40 expression in BMDCs. BMDCs (2 × 10^5^) were stimulated with DMSO (0.01%) (None), inotodiol (25 µM), lanosterol (25 µM), or lanosteral (25 µM) for 24 h. The expressions of CD86 **(A)**, MHC-II **(B)**, and CD40 **(C)** were measured by flow cytometry. Data are presented as means ± SDs (*n* = 3). Statistical significance was tested between two different conditions.

Supplementary Figure 3 Cytokine production by inotodiol-treated peritoneal macrophages and Raw264.7 cells. Peritoneal macrophages **(A)** and Raw264.7 cells **(B)** (2 × 10^5^) were treated with DMSO (0.01%) (None), inotodiol (25 μM), or LPS (1 µg/mL) for 24 h (*n* = 3). The amount of cytokines or chemokines secreted in culture supernatants was detected using a mouse cytokine array kit **(A)** and measured by ELISA **(B)**. Data are presented as means ± SDs (*n* = 3). **p <*0.001, versus None.

Supplementary Figure 4 Effects of wortmannin on CD86 expression and GSK-3β phosphorylation in SB216763-treated BMDCs. **(A)** BMDCs (2 × 10^5^) were pretreated with (+Wortmannin) or not (-Wortmannin) with wortmannin (100 nM) for 1 h and then cultured with DMSO as vehicle control (0.01%) (None), inotodiol (25 µM), LPS (1 µg/mL), or SB216763 (35 nM and 5 µM) for 24 h (*n* = 3). CD86 expression was measured by flow cytometry. **(B)** BMDCs (2 × 10^6^) were pretreated with (+Wortmannin) or not (-Wortmannin) with wortmannin (100 nM) for 1 h and then stimulated with DMSO (0.01%) (None), inotodiol (25 µM), LPS (1 µg/mL), or SB216763 (35 nM and 5 µM) for 20 min. The samples were then subjected to western blot analysis using anti-p-GSK-3β_ser9_ and anti-GSK-3β antibodies. Protein expression of p-GSK-3β was quantified by densitometry and standardized to GSK-3β using ImageJ. Data are the means ± SDs of three independent experiment. **P <*0.05, versus None ***P <*0.05, versus without wortmannin.
