# Supplementary figures and images for: Inotodiol From Inonotus obliquus Chaga Mushroom Induces Atypical Maturation in Dendritic Cells
Source: Front Immunol. 2021 Mar 12;12:650841. doi: 10.3389/fimmu.2021.650841 (PMC7994266; doi:10.3389/fimmu.2021.650841)

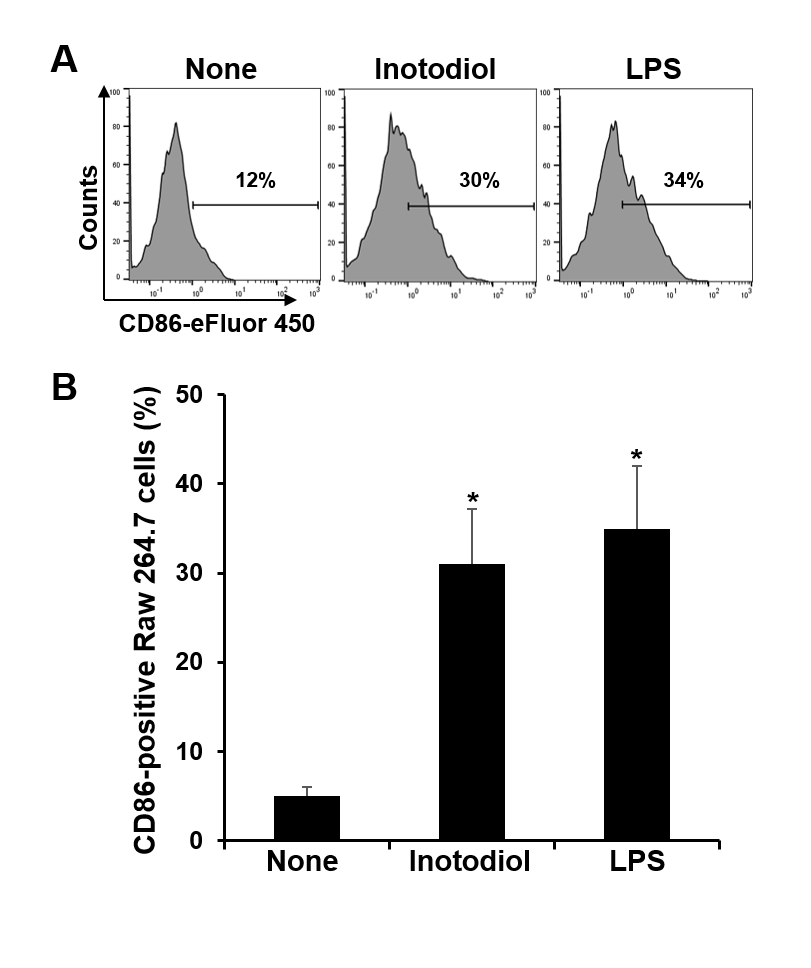

Supplement: Supplementary Figure 1 — Inotodiol increases CD86 expression in macrophages and Raw264.7 cells in vitro. (A) Peritoneal macrophages (2 × 10^5^/mL) isolated from C57BL/6 mice were purified with anti-F4/80 antibody-bound microbeads and treated with DMSO (0.01%) (None), inotodiol (25 μM), or LPS (1 µg/mL) for 24 h. CD86 expression was measured by flow cytometry using an anti-CD86 antibody conjugated with eFluor 450. Representative histograms show CD86 expression in each group (n = 3). (B) Raw 264.7 cells (2 × 10^5^/mL) were treated with DMSO (0.01%) (None), inotodiol (25 μM), or LPS (1 µg/mL) for 24 h. CD86 expression was determined by flow cytometry. Data are presented as means ± SDs (n = 3). *p <0.05, versus None. [file Image_1.tif]

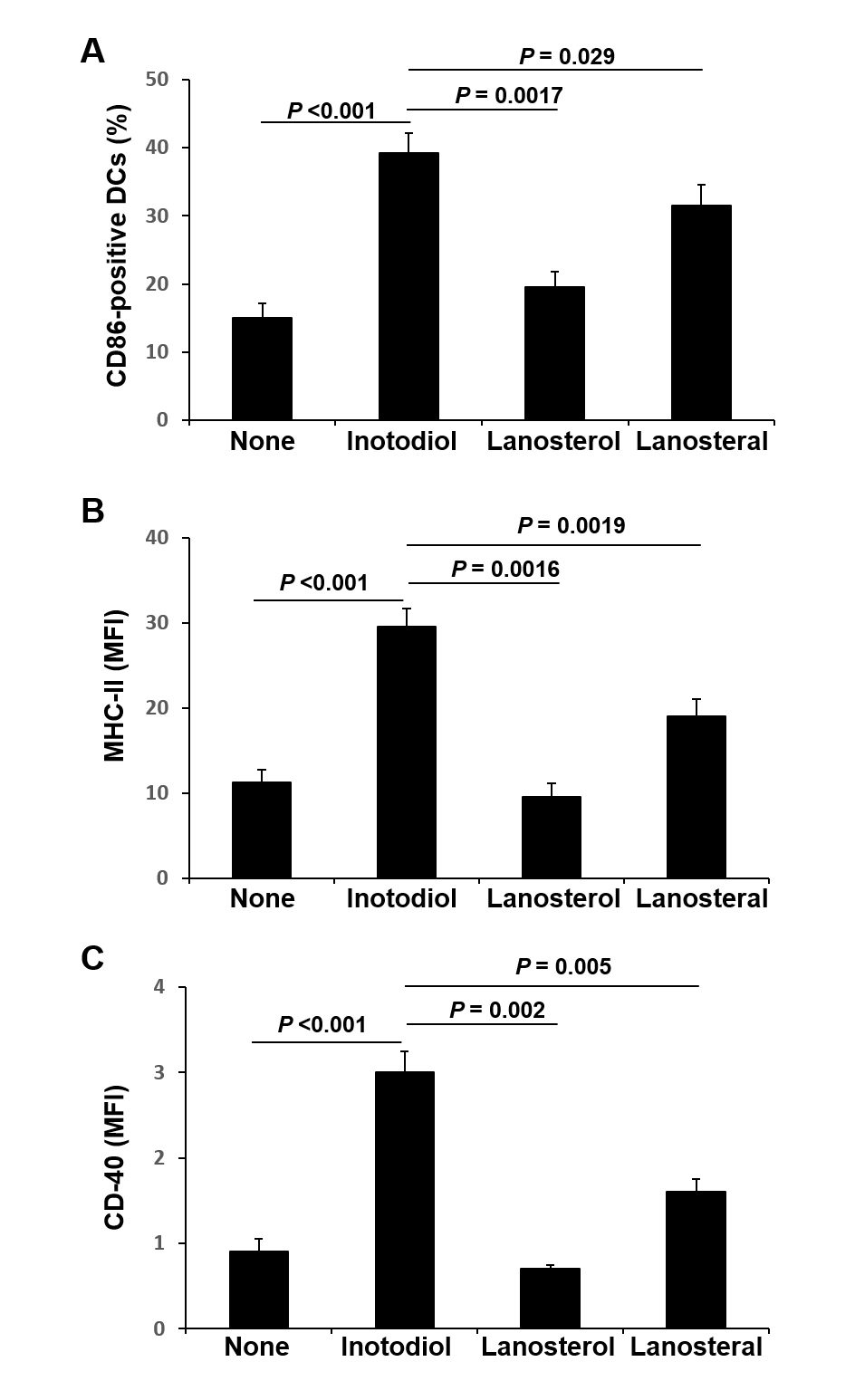

Supplement: Supplementary Figure 2 — Effects of lanosterol, inotodiol, and lanosteral on MHC-II, CD86, and CD40 expression in BMDCs. BMDCs (2 × 10^5^) were stimulated with DMSO (0.01%) (None), inotodiol (25 µM), lanosterol (25 µM), or lanosteral (25 µM) for 24 h. The expressions of CD86 (A), MHC-II (B), and CD40 (C) were measured by flow cytometry. Data are presented as means ± SDs (n = 3). Statistical significance was tested between two different conditions. [file Image_2.tif]

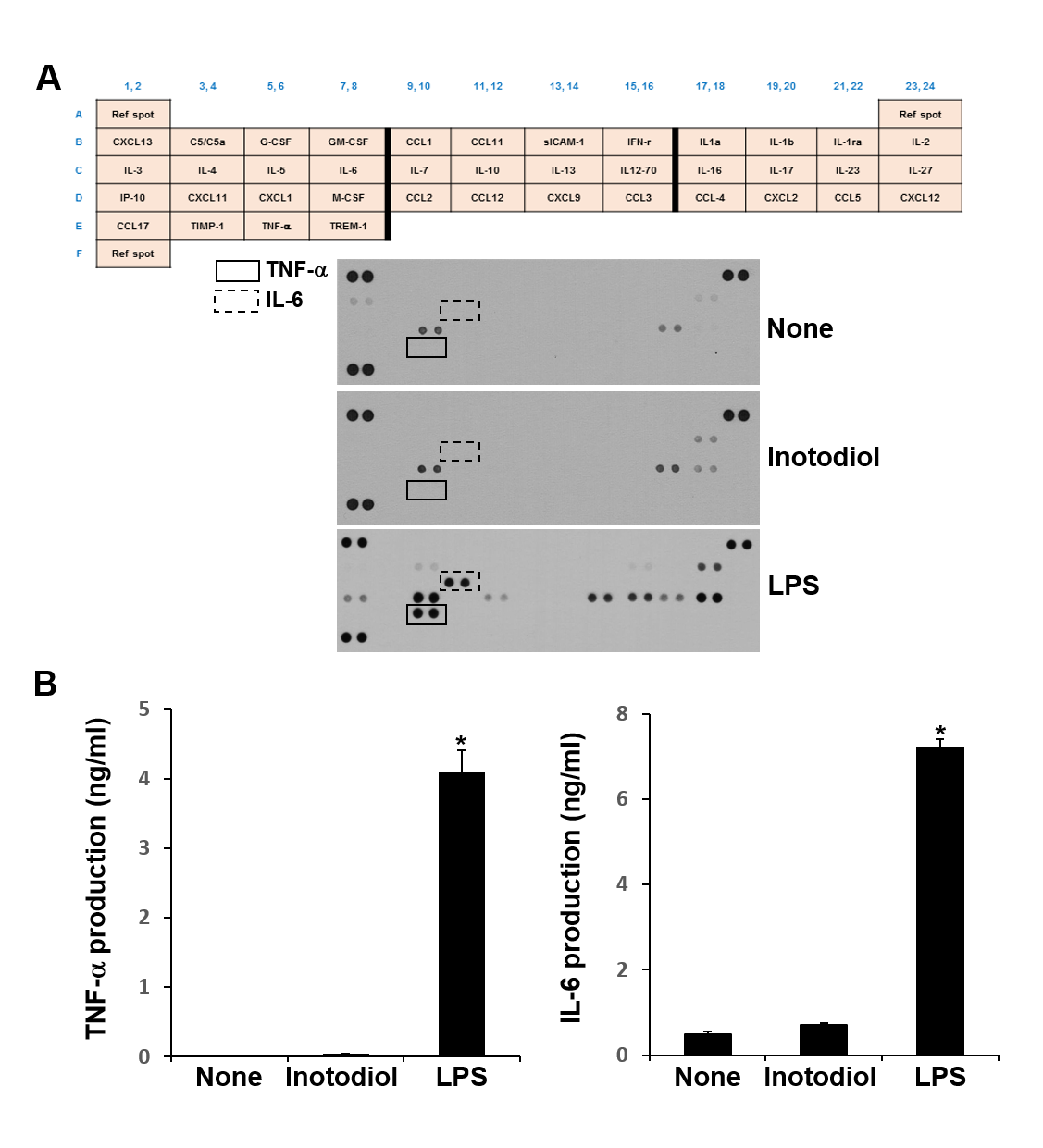

Supplement: Supplementary Figure 3 — Cytokine production by inotodiol-treated peritoneal macrophages and Raw264.7 cells. Peritoneal macrophages (A) and Raw264.7 cells (B) (2 × 10^5^) were treated with DMSO (0.01%) (None), inotodiol (25 μM), or LPS (1 µg/mL) for 24 h (n = 3). The amount of cytokines or chemokines secreted in culture supernatants was detected using a mouse cytokine array kit (A) and measured by ELISA (B). Data are presented as means ± SDs (n = 3). *p <0.001, versus None. [file Image_3.tif]

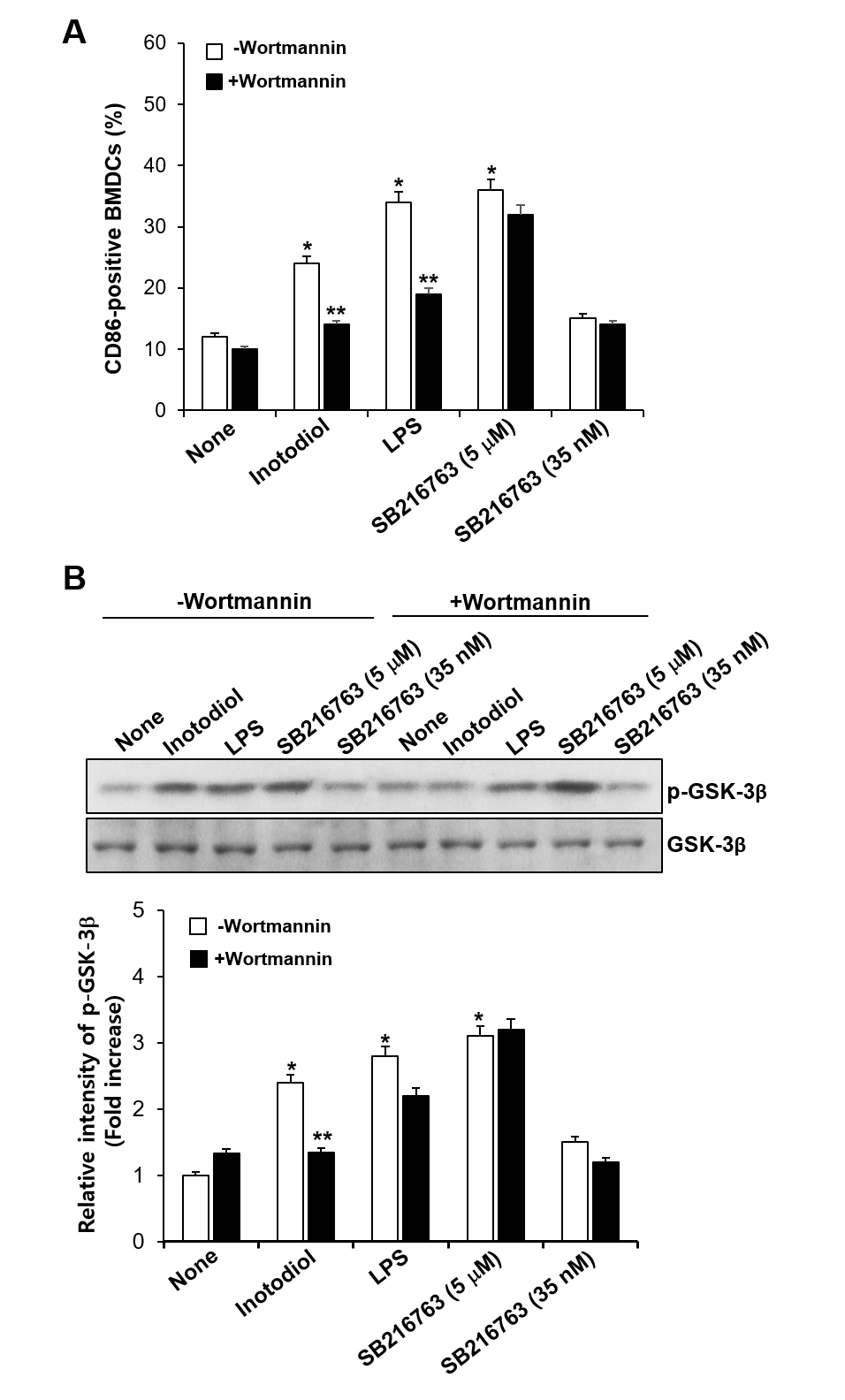

Supplement: Supplementary Figure 4 — Effects of wortmannin on CD86 expression and GSK-3β phosphorylation in SB216763-treated BMDCs. (A) BMDCs (2 × 10^5^) were pretreated with (+Wortmannin) or not (-Wortmannin) with wortmannin (100 nM) for 1 h and then cultured with DMSO as vehicle control (0.01%) (None), inotodiol (25 µM), LPS (1 µg/mL), or SB216763 (35 nM and 5 µM) for 24 h (n = 3). CD86 expression was measured by flow cytometry. (B) BMDCs (2 × 10^6^) were pretreated with (+Wortmannin) or not (-Wortmannin) with wortmannin (100 nM) for 1 h and then stimulated with DMSO (0.01%) (None), inotodiol (25 µM), LPS (1 µg/mL), or SB216763 (35 nM and 5 µM) for 20 min. The samples were then subjected to western blot analysis using anti-p-GSK-3βser9 and anti-GSK-3β antibodies. Protein expression of p-GSK-3β was quantified by densitometry and standardized to GSK-3β using ImageJ. Data are the means ± SDs of three independent experiment. *P <0.05, versus None **P <0.05, versus without wortmannin. [file Image_4.tif]
